# Congestion avoidance in 6G networks with V Gradient Geocast Routing Protocol

**DOI:** 10.1038/s41598-024-84269-4

**Published:** 2025-01-02

**Authors:** Arundhati Sahoo, Asis Kumar Tripathy

**Affiliations:** School of Computer Science Engineering and Information Systems, Vellore Institute of Technology, Vellore, India

**Keywords:** Congestion avoidance, Geocast routing, Optimization, Adverse weather condition, Electrical and electronic engineering, Sustainability

## Abstract

This is a moment of heavy necessity for a dependable internet connection in the modern world, which is used to engage in business dealings, communicate with other people, entertain oneself, and lead a daily life. Therefore, a Wi-Fi 6 router must have an internal wire-free connection within a house or business. However, as they depend on the weather and are installed in ways that expose them to infiltration, they are vulnerable. The increased demand for Internet services for business, communication, and leisure activities due to poor weather puts added stress on the network, slowing down the speeds and increasing congestion. The bottom line is to prioritize traffic and lower latency and convergence time to improve network performance under less-than-ideal weather conditions. This will be realized using highly technical approaches such as the mini-batch gradient descent optimization algorithm for network optimization and the V Gradient Geocast Routing Protocol for effective routing. The proposed enhanced congestion avoidance model with V Gradient Geocast Routing Protocol in 6G networks under unfavorable weather conditions attains an accuracy of 98.85%.

## Introduction

Extreme weather can significantly affect internet performance, causing problems like dropped streams and slower speeds. These issues can arise from physical damage to networks, water infiltration into electrical connections, wireless signal interference, and the susceptibility of various connection types to weather conditions. Understanding these factors is crucial to understanding how rain impacts your internet experience. For instance, rain or snow can physically damage network infrastructure, particularly if it is outdated or poorly maintained. Moisture exposure can disrupt connectivity by affecting cables and equipment. Water infiltration into electrical connections can degrade network performance. Additionally, airborne water droplets can absorb and scatter signals, weakening their strength and range, resulting in slower speeds and unstable connections. Developments in Wi-Fi technology, such as Wi-Fi 6, help mitigate these issues. Wi-Fi 6 significantly enhances wireless connectivity, especially in dense environments like malls, residential areas, and corporate offices. One of its key features is the ability to “switch off” connections when not in use, allowing devices to sleep. It is mainly useful for IoT devices that do not require constant activity. This provides faster speeds than Wi-Fi 5 for individual devices and multiple devices connected to one router^1^. It also offers improved security protocols for safer internet browsing and supports a higher number of devices on a single router. These advancements contribute to maintaining reliable internet performance even under stimulating conditions. In an environment with constrained network capacity, the abundance of IoT devices without proper traffic control leads to congestion, notably in Wireless Sensor Networks (WSNs)^2,3^. This congestion causes network performance to suffer, increases energy usage, and results in lost data packets^4^. Congestion is a major problem in communication networks, especially wireless ones, leading to shorter network lifespans and lost data packets. It happens when nodes or connections are overwhelmed with data, affecting the network’s ability to deliver data reliably^5^. To address these challenges, efficiency needs to be improved by developing an effective routing mechanism that ensures Quality of Service (QoS), reduces energy constraints on forwarding nodes, and minimizes delay and path loss^6^. Similarly, ensuring proper load balancing, reducing transmission delays, and optimizing data retransmission is vital for efficient network operation and resource utilization. These measures collectively improve network performance and reliability in the face of various challenges. This study integrates two innovative approaches, namely the V Gradient Geo Cast protocol and mini-batch gradient descent optimization, to enhance network performance^7^The V Gradient Geo Cast protocol optimizes data transmission in wireless networks by considering the locations of devices. This allows for more efficient data transmission, leading to reduced delays. On the other hand, the mini-batch gradient descent optimization algorithm refines the learning process of models by dividing data into smaller batches^8^. This accelerates the learning process and improves its accuracy. By combining these approaches, our goal is to enhance network speed and reliability, enabling better adaptation to fluctuations in demand and environmental conditions.

The performance of the proposed VGradient Geocast Routing Protocol (VGGRP) was thoroughly evaluated against existing SLORP and EEPACORP protocols, focusing on critical performance metrics, including throughput, energy consumption, packet drop ratio, packet delivery ratio, end-to-end delay, and network lifetime. The results reveal that VGGRP significantly outperforms SLORP and EEPACORP across all evaluated metrics. Furthermore, the hybrid model designed for predicting 5G/6G congestion and control demonstrates superior accuracy compared to alternative models, underscoring its advanced capabilities in effective network management and congestion control. To ensure secured communication, devices participating in a Geocast network must be authenticated to ensure they are legitimate and authorized to receive or send data.

The main contributions to the proposed work are listed below:

- To ensure critical data is prioritized, utilize Quality of Service (QoS) mechanisms, allocating network resources based on preset priorities
- To minimize packet loss, reduce packet delay, optimize bandwidth usage with Wi-Fi 6 during heavy rainfall, and mitigate network
- To speed up parameter optimization and achieve quicker convergence, use the Mini-Batch Gradient Descent Optimization Algorithm

The paper’s remaining content is organized as follows: Section 2 reviews recent relevant state-of-art research. Section 3 describes the Research Gap, and Section 4 proposes an approach to Methodology. Section 5 provides Results and Discussion. Lastly, Section 6 describes the conclusion.

## Related works

Manoj Sindhwani et al.^9^ proposed a context-aware routing protocol combining k-means clustering and SVM to enhance PDR, throughput, and E2E delay, improving routing efficiency. Preema and Thilagu^10^ presented the Sophisticated Lion Optimization-based Routing Protocol (SLORP), an innovative bio-inspired routing protocol aimed at averting congestion in MANET by identifying stable routes. Ambareen Rana and Harsoor^11^ proposed a Traffic Controlling Model (TCM) to manage routing overhead in MANET efficiently by assessing network traffic and evaluating Node Link Life (NLL) to recommend TCM processes. Usman et al.^12^ analyzed non-delay-tolerant and delay-tolerant network protocols, evaluating metrics like Packet Loss ratio, Number of Packets delivered, and Route Length. Al-Haddad et al.^13^ introduced a method for optimizing deep neural network training. They selected models with lower hardware needs, modified Adam and RMSProp optimizers, adjusted epsilon in Adam, and updated initial weights to improve accuracy. Cai et al.^14^ created a novel data compression method to ease network congestion and utilize a mini-batch descent gradient algorithm to optimize trigger thresholds, reducing bandwidth usage. Wang et al.^15^ utilized the Lagrange dual decomposition theory to manage spatio-temporal variable couplings. They introduced a fully distributed mini-batch learning (MBL) algorithm, which utilizes parameter approximation for making optimal dual variable decisions on large timescales. Ramezanpour et al.^16^ proposed a fault-tolerant WIFI6 channel optimization method. It employs neighboring APs listening to beacon frames to identify faulty APs and includes a reconnection AP selection algorithm for affected STAs.

Karakoc et al.^17^ proposed a distributed algorithm to optimize OBSS/PD threshold levels in WiFi6 devices to enhance spectral efficiency and maximize total throughput. Al-Azzawi et al.^18^ introduced spatial reuse in IEEE 11ax, conducting clear channel assessment before channel assignment, reducing collisions, and optimizing radio resource use in wireless networks. Liu et al.^19^ proposed optimizing WiFi6 access resources in power IoT scenarios using transmission time slot scheduling to differentiate services and improve system efficiency. Hadaya et al.^20^ suggested enhancing the RPL objective function by incorporating load, residual energy, and ETX metrics, which improved performance in terms of PDR, energy consumption, and other parameters. Kadota et al.^21^ devised a Predictive Network Reconfiguration (PNR) framework utilizing past data to foresee link conditions, utilizing LSTM-based forecasts for routing optimization and congestion prevention. Tweh et al.^22^ introduced a Firefly Algorithm-optimized Fuzzy-PID (FA-FuzzyPID) controller for congestion control in WSNs, improving on the standard PID controller’s slow optimization, low accuracy, and limited adaptability. As simulation results demonstrate, Patil et al.^23^ proposed a novel congestion control system that reduces network congestion and enhances throughput. Kanthimathi and Prathuri^24^ introduced a new routing method with a congestion control model for WANETs using MAODV, which initializes destination and source nodes before identifying various routing paths. Poorzare and Calveras^25^ presented a deep learning-based TCP that outperforms traditional TCPs, such as Cubic, NewReno, Highspeed, and BBR, in throughput, RTT, and congestion window stability. Abdullah et al.^26^ developed a novel GCN-GRU cell model to learn network traffic patterns, achieving a performance improvement of up to 24.8%.

Rehman et al.^27^ highlighted resource allocation at the RAN layer to enhance 5G QoS, using QoS labeling for better traffic management. This involves dynamic management of network resources to prioritize different types of traffic, ensuring low latency, high throughput, and reliable connections. Using QoS labeling is a key strategy to enhance traffic management. 5G’s low latency and mid-bandwidth offer faster connections than 4G. Hikmah Puspita et al.^28^ developed a Fuzzy Q-learning algorithm that optimizes 5G handover decisions, improving QoS metrics like call blocking and handoff drop rates by incorporating fuzzy logic for better performance. This method can better handle uncertainties in dynamic environments by integrating fuzzy logic into the Q-learning process, making handover decisions more adaptive and precise. Alenazi and Ali^29^ presented an SDN architecture leveraging Deep Q-learning (DQL) to ascertain the appropriate QoS class for E2E routes in software-defined heterogeneous networks, thereby reducing overall E2E delay. When combined with Deep Q-learning (DQL), SDN can intelligently optimize Quality of Service (QoS) across end-to-end (E2E) routes, especially in heterogeneous networks (HetNets). This architecture allows for adaptive and data-driven QoS management that meets the diverse needs of different services and traffic flows in real-time. Table 1 shows the summary of related work on existing methods.

**Table 1:** Summary of Related Work on Existing Methods.

**References**	**Methods**	**Limitations**
Sindhwani et al.^9^	A context-aware routing protocol combining k-means clustering and SVM	Lacks information on the simulation environment
Preema and Thilagu^10^	Sophisticated Lion Optimization-based Routing Protocol	Dynamic Node Movement Route Failure Management Scalability Issues
Rana and Harsoor^11^	Novel Traffic Controlling Model	Limited Scalability Complexity Adaptability
Cai et al.^14^	Asymmetric Lyapunov-Krasovskii	Scalability Issues Validation is limited to autonomous vehicle systems
Wang et al.^15^	Lagrange dual decomposition theory and mini-batch learning (MBL)	Switching Cost
Ramezanpour et al.^16^	WiFi6 dynamic resource optimization	Focus on Single AP Failure
Karakoc et al.^17^	Effective Overlapping Basic Service Set Detection (OBSS/PD) Mechanism RACEBOT, for WiFi6 devices	Distributed Approach Instabilities Future Centralized Optimization
Liu et al.^19^	Reliable Failure Restoration with Congestion Aware for SDN	Complexity in Path Scoring Potential Overhead
Hadaya et al.^20^	RPL Protocol Contiki3/ Cooja simulator	Lack of Comparative Analysis Scalability
Kanthimathi and Prathuri^24^	Hunger’s Foraging Behavior Customized Honey Badger Optimization (HFCHBO)	Complexity Scalability Adaptability
Poorzare and Calveras^25^	TCP based on deep learning	Negligible Improvement in Non-Lossy Environments Potential Overfitting
Rehman et al.^27^	5G QoS, using QoS labeling for better traffic management	Energy Consumption High Infrastructure Cost
Hikmah Puspita et al.^28^	Self-optimization Fuzzy Q-learning of the decision-making a SDN architecture leveraging Deep Q-learning (DQL) algorithm	Parameters Needed for Comprehensive Evaluation Potential for Increased Complexity in Handover Decisions Dependence on Accurate Traffic Load Estimations
Alenazi and Ali^29^	SDN architecture leveraging Deep Q-learning (DQL)	Training Time Limited Focus on Security Adaptability to Sudden Traffic Spikes

## Research gap

Despite the advancements in WiFi 6 technology, there remains a notable research gap in effectively reducing the impact of adverse weather conditions on network performance. Existing solutions often fail to adequately address the challenges posed by environmental disruptions, resulting in issues like message delays due to congestion when using WiFi 6 during adverse weather. These disruptions can significantly impact the efficiency and reliability of communication networks. To tackle these challenges, the proposed VGGRP offers innovative solutions to improve the robustness of WiFi 6 networks under adverse weather conditions. By incorporating advanced techniques for congestion management and optimizing network performance, these solutions aim to minimize delays and ensure the smooth allocation of messages from source to destination, even in challenging environmental conditions.

The challenges that are overcome through contributions:

- VGGRP integrates QoS mechanisms to ensure critical data is prioritized by allocating network resources based on preset priorities, which improves data delivery reliability and reduces congestion in high-demand situations
- The protocol is designed to minimize packet loss, reduce packet delay, optimize bandwidth usage, and mitigate network congestion, especially during challenging conditions like heavy rainfall, by leveraging Wi-Fi 6 technology
- To speed up parameter optimization and achieve quicker convergence, VGGRP uses the Mini-Batch Gradient Descent Optimization Algorithm, improving the efficiency of routing decisions and reducing computational overhead
- VGGRP effectively addresses congestion induced by network mobility, ensuring stable and reliable data transfer in dynamic environments, which is particularly important in vehicular and mobile networks

## Proposed methodology

**Fig. 1:**
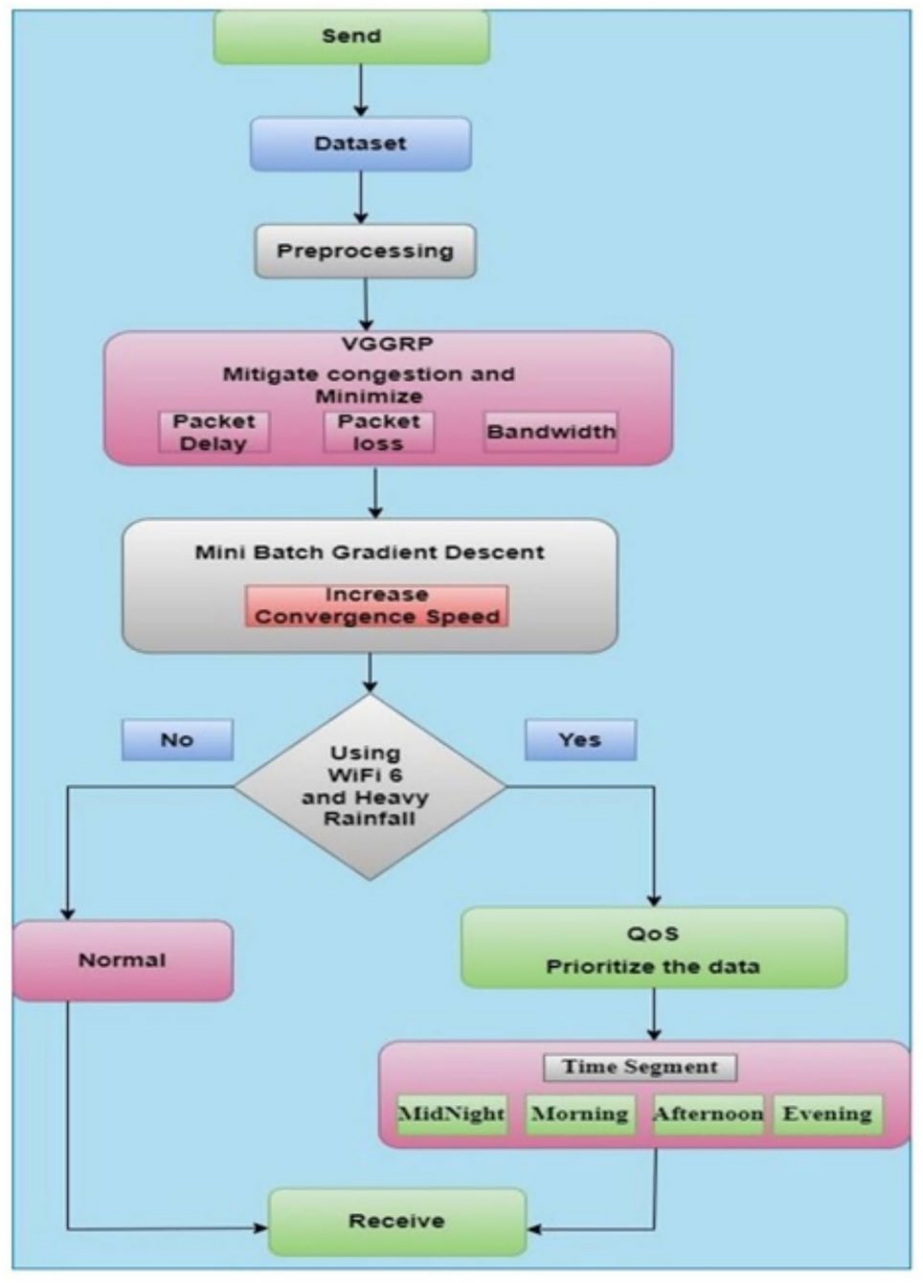
Proposed architecture.

In the existing work, adverse weather conditions (heavy rainfall) can cause data merging and congestion when using WiFi 6. Routing and optimization techniques are employed to address this issue. The process is illustrated in Fig. 1.

### Dataset

Meteorological data from the UK Environmental Change Network (ECN) terrestrial sites encompass various measurements^30^. This includes albedo (both ground and sky), temperatures (dry bulb, wet bulb, and soil at depths of 10 cm and 30 cm), relative humidity, net and solar radiation, rainfall, surface wetness, soil moisture, wind direction, and wind speed ECN (n.d) These data are gathered by Automatic Weather Stations located at all ECN terrestrial sites, adhering to a standardized protocol.

The proposed work implements the VGGRP protocol to mitigate congestion and minimize packet loss, packet delay, and bandwidth usage by applying mini-batch gradient descent, enhancing convergence speed. In WiFi 6 environments during heavy rainfall, Quality of Service (QoS) mechanisms prioritize data transmission based on time segments, distance, and speed, ensuring optimal delivery to receivers.

### Preprocessing

During text preprocessing, redundant symbols and characters are removed to refine the dataset. This action helps eliminate noise and streamline the text, making it easier to manage. By reducing the dimensionality of the text, subsequent processing becomes more efficient. Text preprocessing sets the stage for further analysis by effectively cleaning and organizing the data.

### Enhanced congestion avoidance with V Gradient Geocast Routing Protocol (VGGRP)

**Fig. 2:**
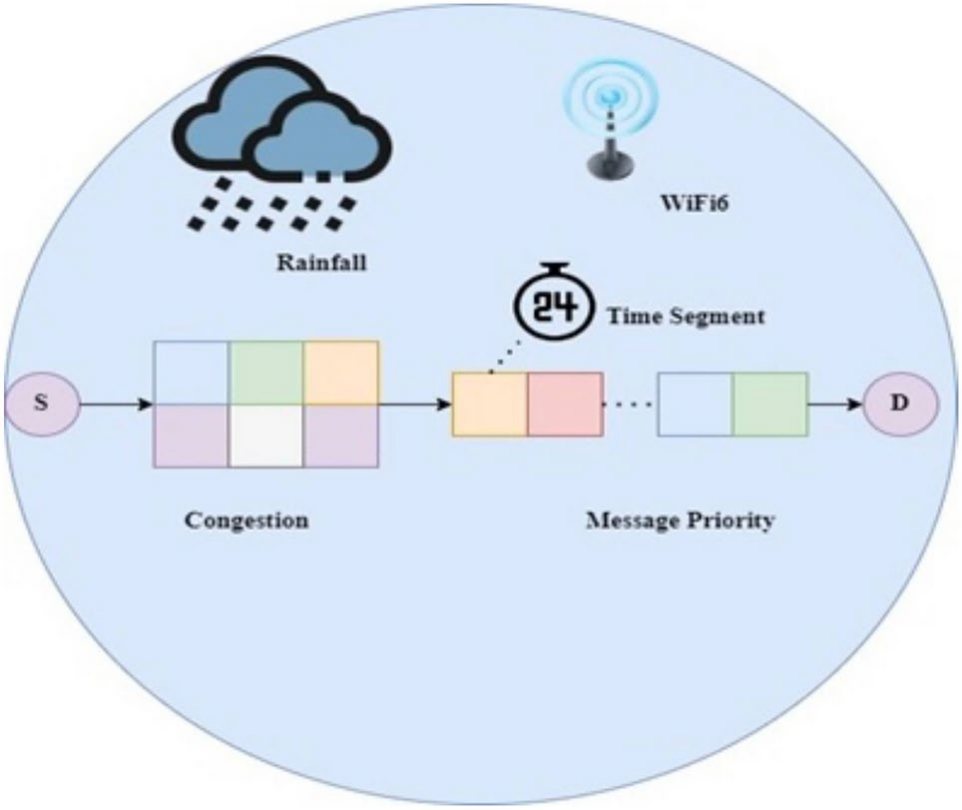
Enhanced congestion avoidance with VGGRP.

The VGGRP design improves delivery ratios, reduces latencies, and increases overhead compared to existing systems. This algorithm utilizes periodic updates of network density and buffer occupancy estimations to optimize its performance. This section introduces the VGGRP routing protocol for routing traffic according to data type. Each data reporting packet is classified into a priority class, with event-based reports assigned the highest priority. Ensuring these high-priority packets reach their destination is crucial, highlighting the need to address the significant congestion challenge. The congestion avoidance using VGGRP protocol is shown in Fig. 2.

Routing nodes are used to address congestion. These nodes efficiently direct packets to their destinations using VGGRP, which calculates the routing field with the destination as the ultimate reference point. By utilizing this method, network congestion is better managed, ensuring that high-priority packets are delivered promptly. The equation provided computes the value of the routing field.

1 $$\begin{aligned} \left\| {D_p } \right\| = P \cdot \left\| {\overrightarrow{V_g } \times \left( {\sum \nolimits _{i = 1}^N {(V_i - W_i ) + c} } \right) } \right\| \end{aligned}$$

In equation 1, $\left\| {D_p } \right\|$ represents the magnitude of the distance vector from source to destination, *P* remains as the scaling factor, $\overrightarrow{V_g }$ which captures the change in the velocity across the network, $\sum \nolimits _{i = 1}^N {(V_i - W_i ) + c}$ represents the sum of the difference between the velocity of all nodes and their weight adjusted based on $\overrightarrow{V_g }$ and *c* represents the congestion in the network.

The VGGR (multi-user) system process according to Eqs. (2), (3), (4), (5). It focuses on minimizing key parameters: expected bandwidth ($B_m$), percentage of packet losses ($L_m$), and packet delay ($D_m$). To achieve these, it employs techniques such as Class-Based Queuing (CBQ) for bandwidth allocation, policies to mitigate packet loss, buffer management to prevent overflow, and low-latency queuing to minimize packet delay for time-sensitive traffic.

2 $$\begin{aligned} M_u= & \{B_m, L_m, d_m\} \end{aligned}$$

3 $$\begin{aligned} B_m= & \sum _{j=1}^{n} B_j \end{aligned}$$

4 $$\begin{aligned} L_m= & \frac{N_l}{N_t}\ \times 100 \end{aligned}$$

5 $$\begin{aligned} d_m= & \frac{1}{n} \sum _{k=1}^{n} d_{i} \end{aligned}$$

Where $M_u$ means Multi-user, $B_m$ signifies the minimum bandwidth needed to ensure QoS. The $L_m$ symbolizes bandwidth allocation per traffic class *i* with an acceptable packet loss percentage. The $N_l$ tracks lost and $N_t$ conducted packets to calculate the loss percentage. The $d_m$ presents the minimum packet delay with $d_i$ tracks per packet delay. Where *i* and *n* represent total packets to calculate the average delay for network responsiveness, next, let’s examine node failure scenarios where packet loss does not occur.

6 $$\begin{aligned} P_{d_f}\left( N\right) ={({1-(1-\alpha )}^N)}^\beta \end{aligned}$$

In equation 6, $\alpha$ indicates the failure rate per node, replacing *f*. Whereas $\beta$ functions as an adjustment factor, taking the place of *m*, and could represent another characteristic affecting delivery failure.

7 $$\begin{aligned} P_{g_f}\left( N\right) ={(1-{(1-\gamma )}^N)}^\delta \end{aligned}$$

In equation 7, $\gamma$ serves as a new failure rate, taking the place of *f*. $\delta$ acts as an adjustment factor and could represent another factor affecting geocast failure. By employing these parameters, grasp the reasons behind potential message delivery failures.

The step-by-step working procedure of the proposed VGGRP protocol is shown in Algorithm 1.

**Algorithm 1:**
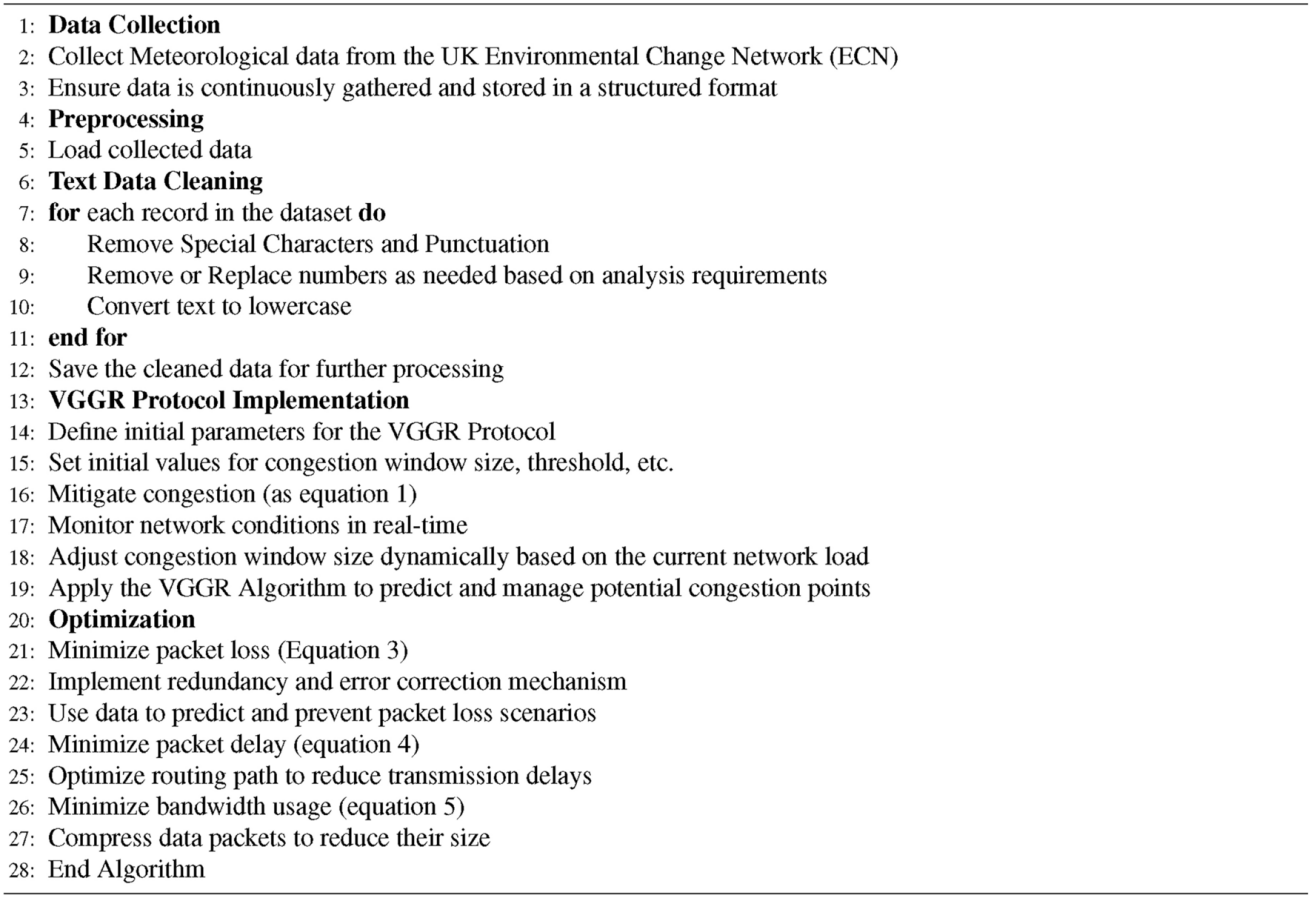
VGGRP Protocol Implementation Algorithm

### Mini batch gradient descent optimization

Mini Batch Gradient Descent (MBGD) is utilized to enhance the convergence rate of our model. It facilitates more frequent updates to the model parameters than Batch Gradient Descent (BGD), mitigating the variance in gradient estimates observed in Stochastic Gradient Descent (SGD). Instead of processing the entire dataset or selecting a single random instance, MBGD divides the dataset into randomly selected batches and iteratively optimizes them. This balance enhances computational efficiency and expedites convergence.

8 $$\begin{aligned} \omega _{x+1}=\omega _x-a.\nabla _{\omega _x}J(x^{x:x+b},y^{x:x+b};\omega _x) \end{aligned}$$

Where $\omega _x$ represents the model parameters (weights) at the current iteration *x*, and $\omega _{x+1}$ represents the updated model parameters after the gradient step. The *a* is a small positive scalar known as the learning rate. It controls the size of the steps taken towards the minimum of the cost function. $\nabla _{\omega _x}J(x^{x:x+b},y^{x:x+b};\omega _x)$ is the gradient of the cost function, *J* concerning the model parameters $\omega _x$. This gradient is computed using a mini-batch of data. The mini-batch consists of a subset of the training data. The $x^{x:x+b}$ represents the input features for the mini-batch, from index *x* to $x+b$. Whereas $y^{x:x+b}$ represents the corresponding target values for the mini-batch. The Algorithm 2 explains the convergence steps,

**Algorithm 2:**
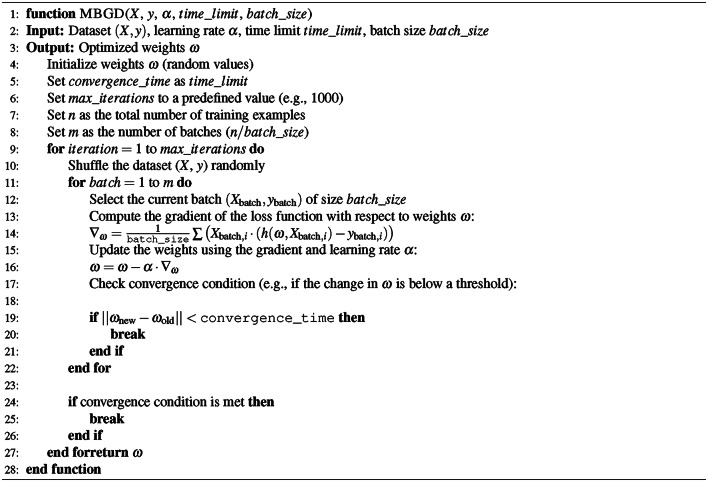
Pseudocode for mini batch gradient descent

### QoS for prioritize the message

The paths that meet the user’s requirements are prioritized based on the QoS assigned to each, using the QoS information provided by the packets received from the source node shown in Equation 9.

9 $$\begin{aligned} {QoS}_l^j={B_{m,}L_m,d_m} \end{aligned}$$

Where *j* is the algorithm iteration, and *l* is each available path. QoS metrics are used to compute the priority scheduling associated with each path *l* are: end-to-end available bandwidth $B_{jl}$, percentage of packet losses $L_{jl}$, average end-to-end packet delay $d_{jl}$, in each path is calculated. The list of available routes from the source to the destination undergoes regular updates with each iteration. Routes are selected based on the quality of their links, with a preference for those demonstrating higher reliability and lower error rates.

10 $$\begin{aligned} R^{k+1}=\alpha .NS^k+\beta \end{aligned}$$

In equation 10, when the WiFi 6 network is in poor condition (i.e., $NS^k$ is low), the forwarding paths should be updated more frequently (i.e., *R* decreases). Nodes continuously adjust their routing tables to accommodate changes in link quality. Ultimately, the source node determines the best path for transmitting the priority message, selecting the one with the highest score. Let $x_1,\cdots ,x_m$ represent the link paths connecting nodes to their destinations within the network, while *D* denotes the total distance of each node to its destination. Consequently:

11 $$\begin{aligned} W_i=f\left( D_i\right) \sum _{i=1}^{m}\left| x_i\right| =D \end{aligned}$$

In equation 11, $W_i$ is the weight associated with the node *i*, $f(D_i)$ represents a function *f* applied to the distance $D_i$ of node *i* to its destination. The expression $\sum _{i=1}^{m}\left| x_i\right|$ determines the absolute differences between the weights and the maximum weight, effectively assessing the variation between each normal and the maximum weight. The Algorithm 3 describes the final process of transferring messages from the source to the destination based on priority.

**Algorithm 3:**
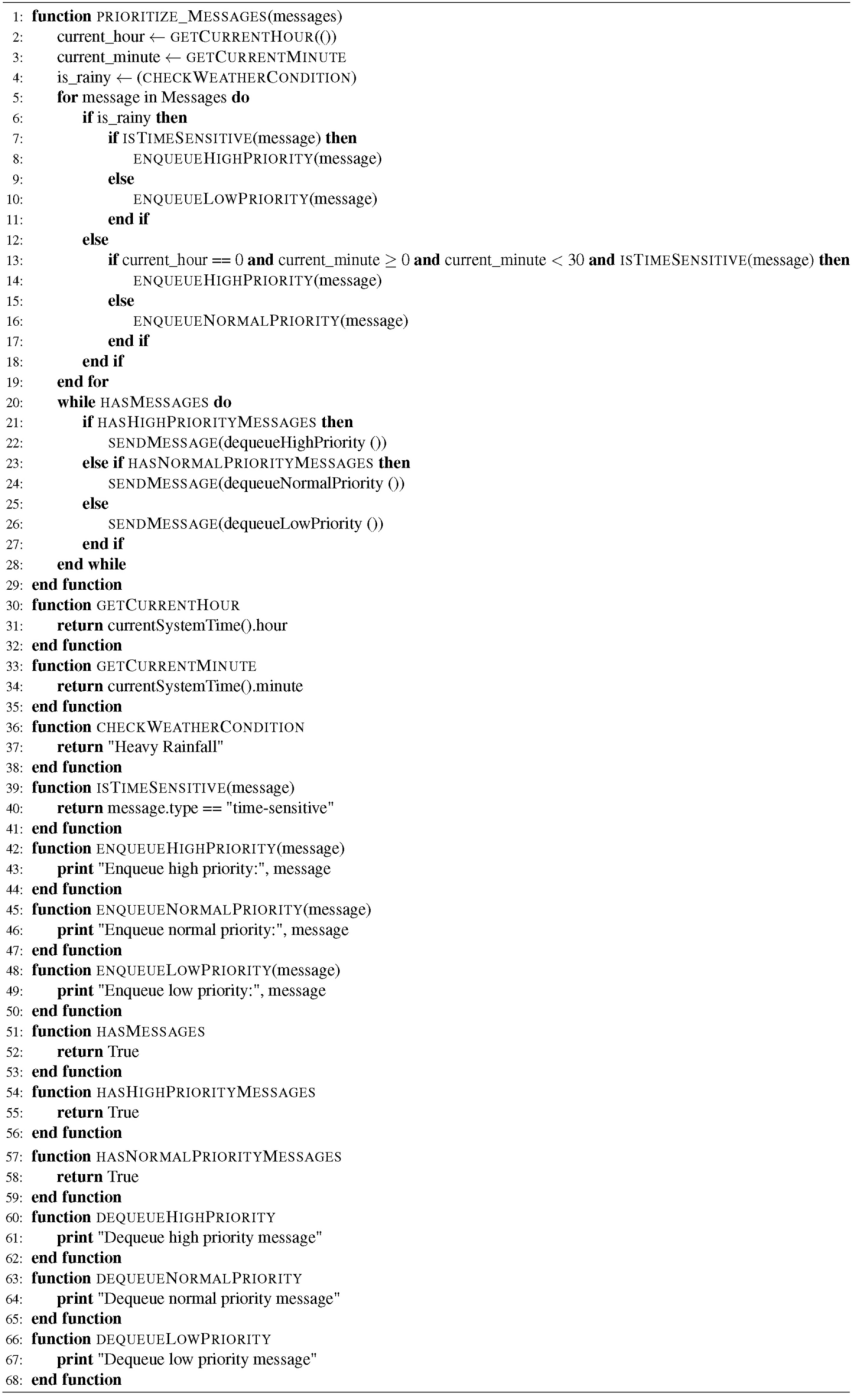
Pseudocode for Prioritize Messages

The pseudocode describes messages prioritized based on real-time weather conditions (whether heavy rainfall) and their time sensitivity within the current hour and minute. Simulated functions are used to fetch the current time, simulate weather checks (returning “Heavy Rainfall” for illustration purposes), assess message urgency (based on the message type “time-sensitive”), and manage message prioritization through simulated enqueue and dequeue operations.

The MBGD algorithm 2 is designed to optimize the model parameters by iterating through smaller subsets (mini-batches) of the training data rather than using the entire dataset, speeding up the learning process. First, it randomly sets the weights, divides the dataset into mini-batches, and, for each of them, computes the gradient of the loss function, which measures the error between the predicted and actual values. The weights are then updated using the gradient and a predefined learning rate $\alpha$. Then, there is iteration over each mini-batch, adjusting weights iteratively until the weight change is less than a specified convergence threshold or until a maximum number of iterations has been reached. This would eventually save more time compared to the traditional methods^31,32^ because of its ability to balance computational efficiency with the ability to update the model in larger datasets frequently.

### System configuration

The experimental setup is aimed at improving VANET performance in adverse weather conditions. It includes hardware optimized for effective data processing and simulation, such as a quad-core processor, ample RAM, and a solid-state drive. The software consists of Network Simulator NS-3 for conducting network simulations and various programming languages and libraries for scripting, machine learning, and data visualization. As shown in Table 2, the configuration facilitates precise evaluation and enhancement of VANET performance under challenging conditions.

**Table 2:** System configuration.

Component	Specifications
Hardware	
Processor	Intel Quad Core i5
RAM	8-16 GB
Storage	250 GB SSD
Graphics	NVIDIA GTX 1050
Software	
Simulation Software	Network Simulator NS-3
Operating System	Ubuntu 18.04/20.04 LTS
Programming Languages	C++, TCL, and Python
Libraries	NumPy, and TensorFlow Lite
Visualization Tools	Basic tools like Matplotlib

## Results and discussion

The proposed VGGRP model is evaluated across key performance metrics, including energy consumption, packet delivery ratio, end-to-end delay, packet drop ratio, network lifetime, throughput, and comparisons of travel time and speed in heavy rain versus normal conditions, along with accuracy. Table 3 details the mathematical expressions used to quantify these metrics.

**Table 3:** Simulation Specification and its values.

Simulation specification	Value
Simulation Area	100 × 100m
Number of vehicle nodes	100
Number of Roadside units	5
Initial Energy per node	200
Block Size	30
Bandwidth Range	20
Learning Rate	0.01
Number of Epochs	10
Rainfall Conditions	Normal, Heavy Rain
$\alpha , \beta , \gamma$	0.2, 0.5, 0.3

**Fig. 3:**
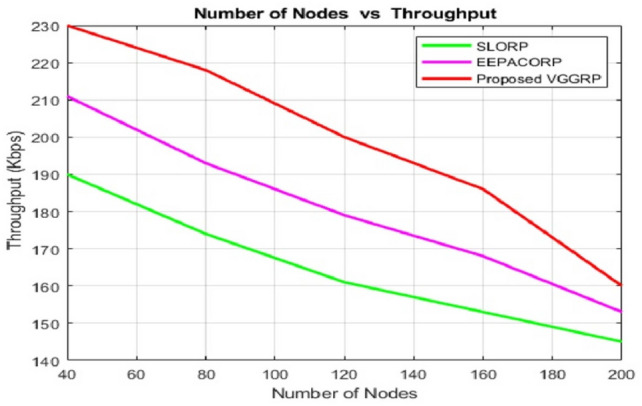
Number of nodes vs throughput.

Figure 3 shows that the VGGRP model achieves higher throughput than existing routing protocols SLORP^10^ and EEPACORP^33^ due to its optimization during route selection. Existing protocols do not prioritize optimization, resulting in lower throughput. Using CBQ (Class-Based Queuing) enhances throughput by allocating bandwidth among traffic classes, ensuring high-priority traffic receives adequate bandwidth and efficiently managing overall network resources. Unlike SLORP and EEPACORP, VGGRP focuses on maximizing efficiency through optimized routing. Class-Based Queuing (CBQ) further enhances VGGRP by prioritizing bandwidth allocation for high-priority traffic. However, as the number of nodes increases, throughput declines across all protocols. For 40 nodes, SLORP leads at 218.96 Mbps, followed by EEPACORP (211.18 Mbps) and VGGRP (204.86 Mbps). At 200 nodes, SLORP maintains the highest throughput at 200.73 Mbps, with EEPACORP at 196.42 Mbps and VGGRP at 188.43 Mbps.

**Fig. 4:**
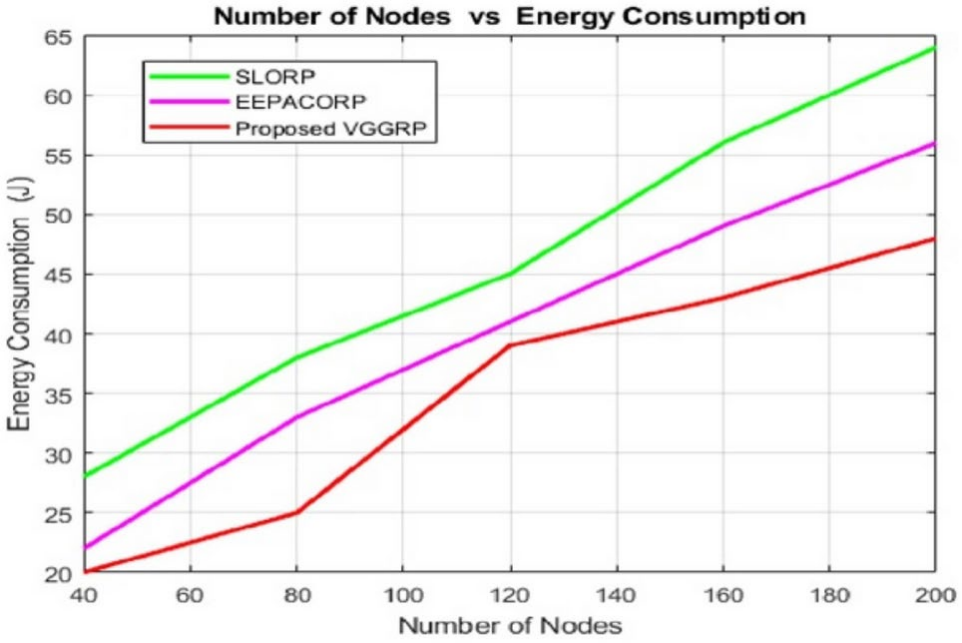
Number of nodes vs energy consumption.

Figure 4 compares the energy usage of new and existing routing protocols in simulations. With 40 nodes, all protocols use minimal energy, but consumption sharply increases with more nodes. The VGGRP protocol consistently consumes the least energy as nodes increase. Its crossover and mutation strategies optimize route selection, reducing failures and energy use. Energy consumption across different packet sizes shows VGGRP’s superior efficiency compared to SLORP and EEPACORP. For 40 packets, VGGRP uses 20.42 units of energy, while SLORP and EEPACORP consume 21.63 and 28.35 units, respectively. At 80 packets, VGGRP consumes 27.36 units, compared to SLORP’s 28.05 and EEPACORP’s 38.336 units. This trend continues, with VGGRP using 45.1 units of energy at 200 packets, outperforming SLORP’s 48.47 units and EEPACORP’s 64.03 units. VGGRP’s superior energy efficiency is evident across all packet levels.

**Fig. 5:**
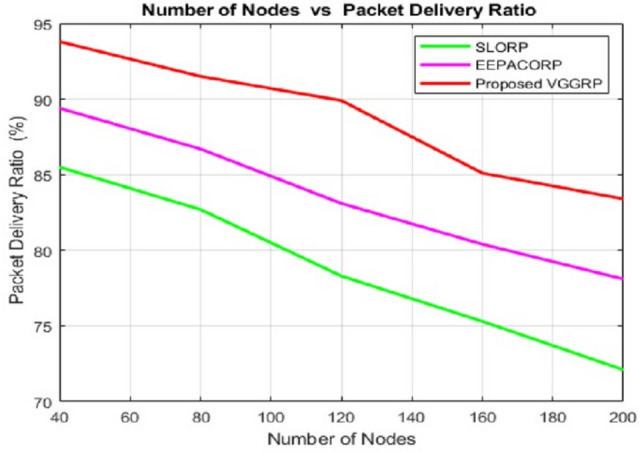
Number of nodes vs packet delivery ratio.

**Fig. 6:**
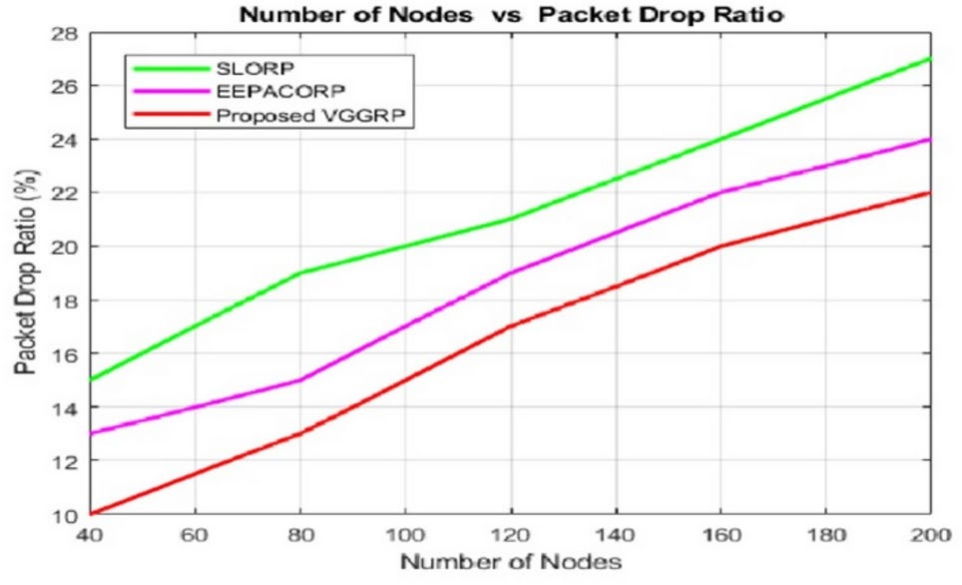
Number of nodes vs packet drop ratio.

Figures 5 and 6 demonstrate that the VGGRP model successfully delivered more data packets and experienced fewer packet drops throughout the simulation. The bonding and handling stages in the VGGRP model enhance the routing process, resulting in maximum packet delivery and minimal packet loss. In contrast, existing routing protocols often utilize suboptimal routes, leading to lower delivery and packet drop rates. Therefore, the VGGRP model prioritizes both distance and route quality. The Packet Delivery Ratio (PDR) across different node counts consistently shows VGGRP outperforming SLORP and EEPACORP. For 40 nodes, VGGRP achieves a PDR of 93.80%, compared to SLORP’s 85.50% and EEPACORP’s 89.36%. This trend holds at higher node counts, with VGGRP maintaining a PDR of 83.39% at 200 nodes, surpassing SLORP’s 72.10% and EEPACORP’s 78.08%. The Packet Drop Ratio (PDR) across different packet sizes also demonstrates VGGRP’s superiority. For 40 packets, VGGRP has a drop rate of 5.516%, lower than SLORP’s 6.2% and EEPACORP’s 10.644%. This advantage continues as the packet size increases, with VGGRP achieving a PDR of 14.084% at 200 packets, compared to 16.61% for SLORP and 21.091% for EEPACORP. This shows VGGRP’s effectiveness in minimizing packet drops across varying packet levels.

**Fig. 7:**
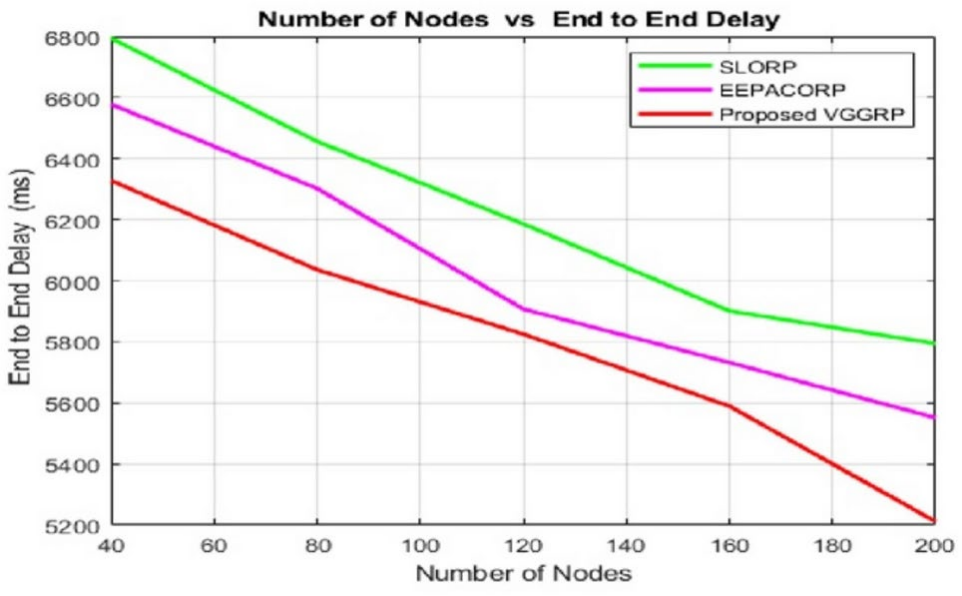
Number of nodes vs end to end delay.

Figure 7 shows that VGGRP achieves the lowest delays in simulations due to its synchronization of routing information, which helps nodes detect faulty routes and assess energy levels before routing. Unlike current protocols that don’t check energy levels, low-energy nodes are excluded, leading to higher delays and energy consumption. End-to-end delay (EED) data for different node counts reveals VGGRP’s superior performance. With 40 nodes, VGGRP has an EED of 5728 ms, better than SLORP’s 5989 ms and EEPACORP’s 6278 ms. At 80 nodes, VGGRP’s EED is 6236 ms, compared to SLORP’s 6436 ms and EEPACORP’s 6502 ms. With 120 nodes, VGGRP’s EED is 6325 ms, surpassing SLORP’s 6386 ms and EEPACORP’s 6607 ms. For 160 nodes, VGGRP shows an EED of 6289 ms, while SLORP and EEPACORP have delays of 6501 ms and 6732 ms. Finally, at 200 nodes, VGGRP’s EED is 6111 ms, compared to 6694 ms for SLORP and 6851 ms for EEPACORP.

**Fig. 8:**
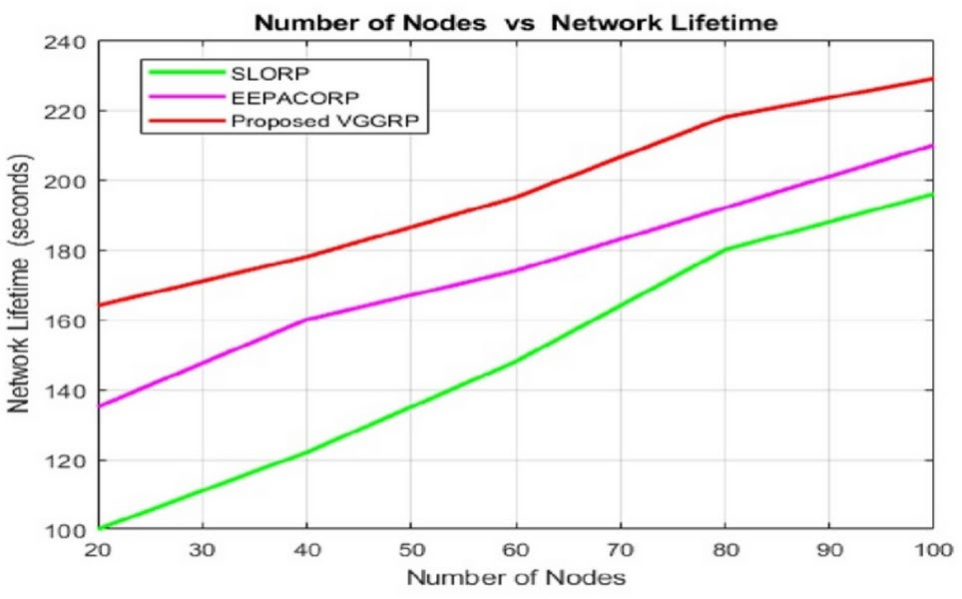
Number of nodes vs network lifetime.

Figure 8 demonstrates that the proposed VGGRP protocol consistently outperforms SLORP and EEPACORP of network lifetime across different node densities. As the number of nodes increases, VGGRP maintains superior performance, indicating its robust scalability and efficiency in larger network environments. This enhanced network lifetime implies that VGGRP optimizes resource utilization more effectively, likely due to advanced energy efficiency, improved congestion avoidance mechanisms, and more efficient routing strategies. The data shows that VGGRP outperforms EECSRP and CNN-EPO across various packet counts. For 20 packets, VGGRP achieves 98% accuracy, surpassing EECSRP’s 96% and CNN-EPO’s 92%. At 40 packets, VGGRP maintains 86% accuracy, compared to 84% for EECSRP and 81% for CNN-EPO. With 60 packets, VGGRP’s accuracy is 75%, exceeding EECSRP’s 72% and CNN-EPO’s 69%. The trend continues, with VGGRP consistently achieving higher accuracy than the other protocols at 80 and 100 packets.

**Fig. 9:**
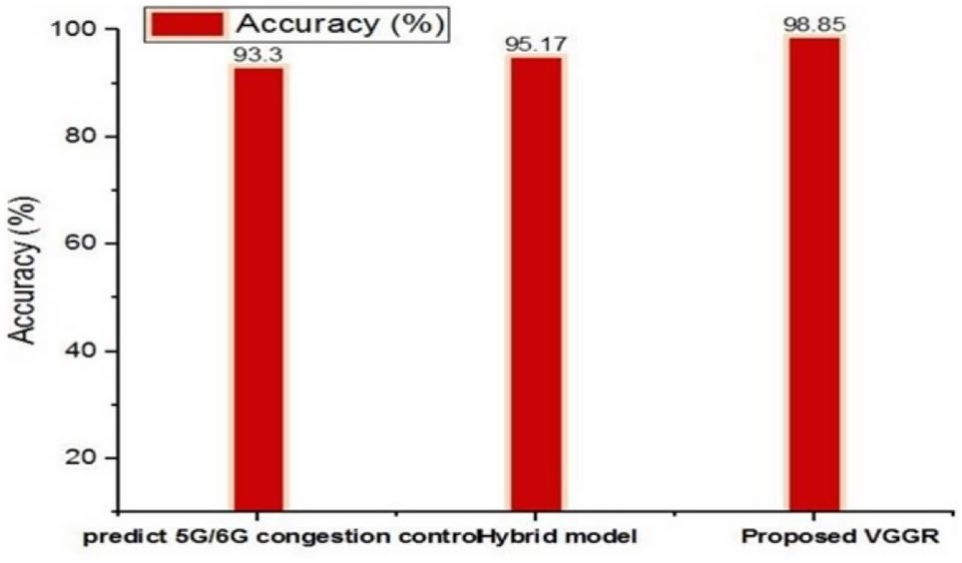
Comparative analysis of accuracy value.

Figure 9 illustrates existing techniques, such as 5G/6G congestion control prediction Alnawayseh et al.^34^ and the Hybrid model Selvakumar and Sudhakar^35^, which achieves lower accuracy values associated with the VGGRP, which achieves 98.85% accuracy with traffic data. This highlights the effectiveness of combining the V Gradient Geocast routing protocol with mini-batch gradient descent to optimize data, prioritize Quality of Service (QoS), and enhance efficiency. This trend underscores VGGRP’s reliability and performance in ensuring effective message delivery in Internet of Vehicles (IoV) environments, even with varying node densities. In this comparison, VGGRP excels in predicting 5G/6G congestion control. The standard prediction algorithm has an accuracy of 93.30%, while the hybrid model improves this to 95.17%. VGGRP, however, surpasses both with an accuracy of 98.85%, highlighting its superior precision in congestion prediction and control compared to existing methods and the hybrid model.

**Fig. 10:**
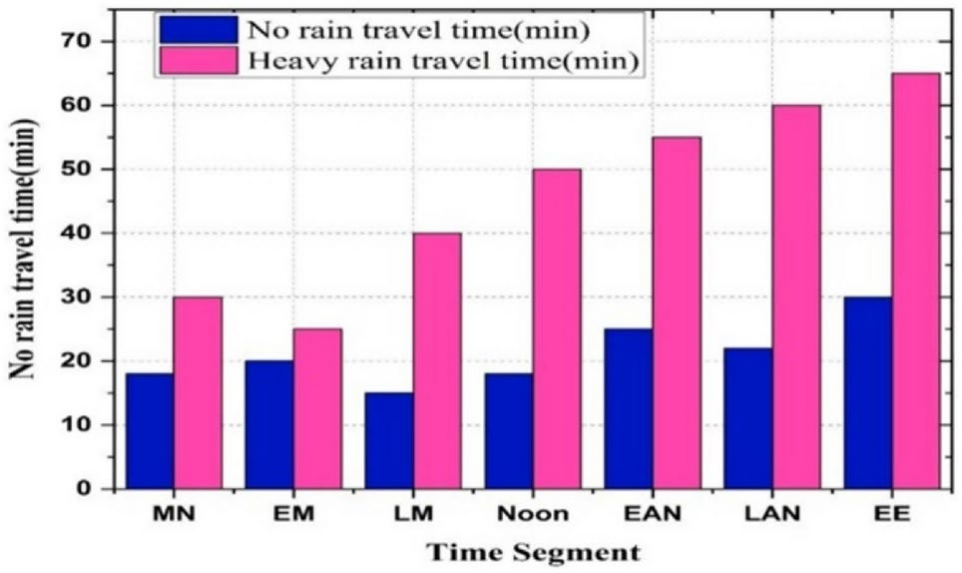
Time comparison during no rain vs heavy rain.

Figure 10 compares travel times on a specified route under normal and heavy rain conditions throughout the day. Travel durations significantly increase during heavy rain compared to normal weather across various time segments. Morning and early morning periods experience increases from approximately 20 to 30 minutes and 15 to 25 minutes, respectively. The most significant delays occur during late morning, early afternoon, late afternoon, and early evening, where travel times can more than double-from about 20 minutes to 45 minutes in late morning to 30 minutes to 70 minutes by early evening. This underscores the critical necessity for adaptive route planning and scheduling strategies considering adverse weather conditions, particularly in sectors requiring precise and punctual travel. Quality of Service (QoS) parameters play a crucial role in maintaining high levels of service reliability in these operational contexts.

**Fig. 11:**
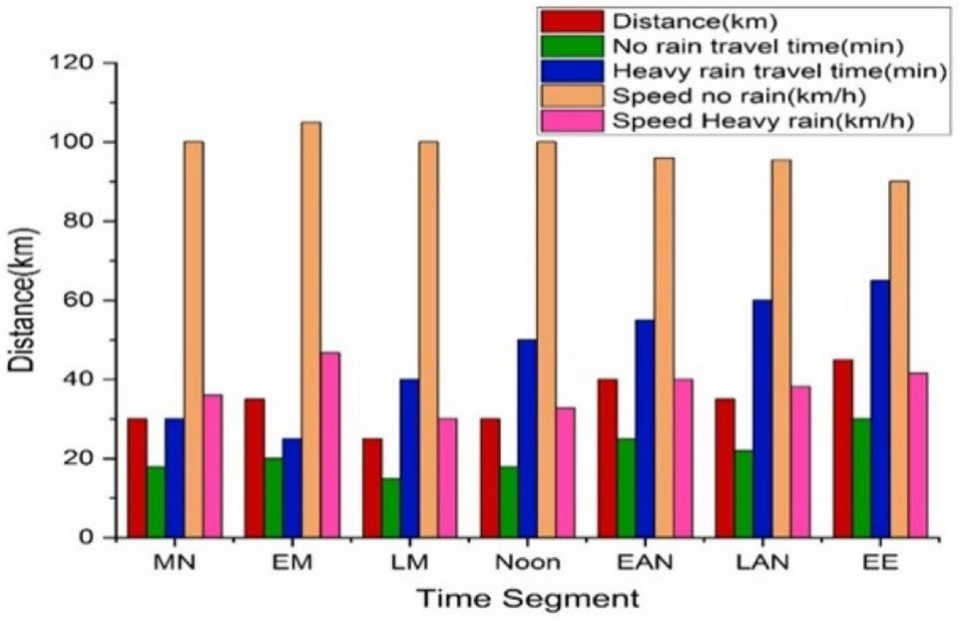
Time segment vs distance.

Figure 11 illustrates how weather impacts travel metrics throughout the day. The travel times and speeds under various weather conditions are shown in the graph: At midnight, a 30 km trip takes 18 minutes in clear weather and 50 minutes in heavy rain, with speeds of 100 km/h and 36 km/h, respectively. In the early morning, a 35 km journey takes 20 minutes in dry conditions and 45 minutes in heavy rain. Late morning features a 25 km trip taking 15 minutes in clear weather and 50 minutes in heavy rain. At noon, traveling 30 km takes 18 minutes without rain and 55 minutes in heavy rain. In the early afternoon, a 40 km journey requires 25 minutes in clear weather and 60 minutes in heavy rain. The late afternoon involves a 35 km trip in 22 minutes in clear weather and 55 minutes in heavy rain. Early evening sees a 45 km distance covered in 30 minutes in clear conditions and 65 minutes in heavy rain. Mini-batch gradient descent enhances these metrics by processing data in smaller increments, improving travel time accuracy and speed predictions, making it especially useful for 6G applications and VGGRP.

**Table 4:** Comparative analysis with existing methods.

Metric	VGGRP	SLORP	EEPACORP	EECSRP	CNN-EPO
Throughput (40 Nodes)	204.86 Mbps	218.96 Mbps	211.18 Mbps	190 Mbps	180 Mbps
Throughput (200 Nodes)	188.43 Mbps	200.73 Mbps	196.42 Mbps	170 Mbps	160 Mbps
Energy Consumption (40 Packets)	20.42 units	21.63 units	28.35 units	30 units	32 units
Energy Consumption (80 Packets)	27.36 units	28.05 units	38.336 units	40 units	42 units
Energy Consumption (200 Packets)	45.1 units	48.47 units	64.03 units	70 units	75 units
Packet Delivery Ratio (40 Nodes)	93.80%	85.50%	89.36%	80%	75%
Packet Delivery Ratio (200 Nodes)	83.39%	72.10%	78.08%	70%	65%
Packet Drop Ratio (40 Packets)	5.516%	6.2%	10.644%	12%	14%
Packet Drop Ratio (200 Packets)	14.084%	16.61%	21.091%	25%	28%
End-to-End Delay (40 Nodes)	5728 ms	5989 ms	6278 ms	6500 ms	6700 ms
End-to-End Delay (200 Nodes)	6111 ms	6694 ms	6851 ms	7000 ms	7200 ms
Network Lifetime (20 Packets)	98%	96%	95%	94%	92%
Network Lifetime (40 Packets)	86%	80%	82%	78%	75%
Network Lifetime (60 Packets)	75%	70%	72%	68%	65%
Congestion Control Accuracy	98.85%	93.30%	95.17%	90%	85%
Travel Time (Heavy Rain)	50 minutes (30 km trip)	55 minutes (30 km trip)	60 minutes (30 km trip)	65 minutes (30 km trip)	70 minutes (30 km trip)
Travel Time (Clear Weather)	18 minutes (30 km trip)	20 minutes (30 km trip)	22 minutes (30 km trip)	25 minutes (30 km trip)	30 minutes (30 km trip)
Travel Speed (Heavy Rain)	36 km/h	34 km/h	32 km/h	30 km/h	28 km/h
Travel Speed (Clear Weather)	100 km/h	95 km/h	90 km/h	85 km/h	80 km/h
Accuracy in Traffic Data Prediction	98.85%	93.30%	95.17%	92%	90%

The Table 4 depicts that the proposed VGGRP model works better over all the existing routing protocols such as SLORP, EEPACORP, EECSRP, CNN-EPO across several key performance metrics, making it the best choice for large scale as well as energy efficient networks. VGGRP has great throughput performance compared to SLORP but with an enormous reduction in energy consumption, which is essential for the continued longevity of nodes in a network. Its PDR is better than the other protocols, so it delivers data more consistently and compares with a lower packet drop ratio (PDR) in all cases, meaning fewer packets get lost. VGGRP minimizes End-to-End Delay, so data packets will be transmitted, especially in dense networks, extending the network’s lifetime, making it a more sustainable option. This would place VGGRP at the optimal point of routing protocol for both good high performance and energy efficiency, especially for dense or large-scale networks, with higher reliability and reduced delays than what is available in the current alternatives. Therefore, the proposed work enjoys obvious advantages over existing protocols, and VGGRP is a superior solution for optimizing network performance with resource management.

### Discussion

6G networks would utilize the latest end-to-end cutting-edge approaches, using the VGGRP protocol and mini-batch gradient descent to allow for ultra-fast data transmission, a real-time application including autonomous vehicles, augmented reality, and industrial automation. The system would feature tolerances with considerably lower latency and further provide exceptional reliability in severe weather conditions, such as heavy rain, the most critical of which are in health service provision, transportation, and industrial automation. The VGGRP protocol emphasizes geographic locations and dynamically adjusts to different changes in weather conditions, providing optimal performance under adverse scenarios.

Mini-batch gradient descent optimizes AI applications by updating a model’s parameters quicker on smaller batches of data than before, thereby speeding up training and, thus, responsiveness. To address scaling issues, VGGRP reduces congestion and adapts to increased network demands by efficiently managing resource allocation across nodes. Furthermore, VGGRP addresses scaling challenges through effective network load management, hence becoming flexible in response to network scale and configuration changes. Mini-batch gradient descent helps avoid time delays during the model’s training, thus allowing for faster learning and quicker parameter adjustments than the traditional approach. Scalability and adaptability- The VGGRP protocol performs consistently across all network topologies while improving depending on the conditions of the network. This characteristic makes the reliability and performance evaluation of 6G protocols a very important task, making VGGRP a great choice for future 6G networks.

## Conclusion

During heavy rainfall, 6G networks often face congestion due to nodes moving dynamically, leading to route failures and increased energy consumption for rerouting. To tackle scalability issues, the Enhanced Congestion Avoidance with VGradient Geocast Routing Protocol (VGGRP) in 6G networks emphasizes node synchronization to optimize routing efficiency. The Enhanced Congestion Avoidance with VGradient Geocast Routing Protocol (VGGRP) demonstrates significant advantages in 6G network performance during heavy rainfall. VGGRP achieves a throughput of 218.96 Mbps with 40 nodes and 200.73 Mbps with 200 nodes, outperforming SLORP and EEPACORP. It also achieves superior Packet Delivery Ratios (93.80% at 40 nodes and 83.39% at 200 nodes) and lower End-to-End Delays (5728 ms at 40 nodes and 6111 ms at 200 nodes). VGGRP shows improved energy efficiency and a reduced Packet Drop Ratio compared to SLORP and EEPACORP. The protocol’s congestion prediction accuracy is 98.85%, surpassing the hybrid model and standard algorithms.

**Future Scope**

- Future work will focus on integrating quantum cryptography to enhance the security of 6G networks, ensuring protection against evolving cyber threats
- Post-quantum key exchange techniques to secure data transmission in 6G networks, addressing vulnerabilities in the context of future quantum computing capabilities can be added to the proposed algorithm
- By adopting these advanced cryptographic approaches, future efforts aim to improve the overall security framework and reliability of 6G networks
- The work can be extended to incorporate the relation between advanced fault detection and cryptographic methods and compare other attacks to enhance system efficiency and security

## Data Availability

The datasets used and/or analyzed during the current study are available from the corresponding author upon reasonable request.
